# Low immediate scientific yield of the PhD among medical doctors

**DOI:** 10.1186/s12909-016-0713-2

**Published:** 2016-07-24

**Authors:** Emil L. Fosbøl, Philip L. Fosbøl, Sofie Rerup, Lauge Østergaard, Mohammed H. Ahmed, Jawad Butt, Julie Davidsen, Nirusiya Shanmuganathan, Simon Juul, Christian Lewinter

**Affiliations:** Department of Cardiology B, University Hospital of Copenhagen, Rigshospitalet Blejdamsvej 9, 2100 København Ø, Denmark; The Technical University of Denmark, Lyngby, Denmark

**Keywords:** Observational study, Danish PhD schools, Medical degree, PhD, Peer-reviewed publications, Medicine, Surgery

## Abstract

**Background:**

We studied the scientific yield of the medical PhD program at all Danish Universities.

**Methods:**

We undertook a retrospective observational study. Three PhD schools in Denmark were included in order to evaluate the postdoctoral research production over more than 18 years through individual publications accessed by PubMed.

**Results:**

A total of 2686 PhD-graduates (1995–2013) with a medical background were included according to registries from all PhD schools in Denmark. They had a median age of 35 years (interquartile range (IQR), 32–38) and 53 % were women at the time of graduation. Scientific activity over time was assessed independently of author-rank and inactivity was measured relative to the date of graduation. Factors associated with inactivity were identified using multivariable logistic regression. 88.6 % of the PhD theses were conducted in internal medicine vs. 11.4 % in surgery. During follow-up (median 6.9 years, IQR 3.0–11.7), PubMed data searches identified that 87 (3.4 %) of the PhD graduates had no publication after they graduated from the PhD program, 40 % had 5 or less, and 90 % had 30 or less. The median number of publications per year after PhD graduation was 1.12 (IQR 0.61–1.99) papers per year. About 2/3 of the graduates became inactive after 1 year and approximately 21 % of the graduates remained active during the whole follow-up. Female gender was associated with inactivity: adjusted odds ratio 1.59 (95 % confidence interval 1.24–2.05).

**Conclusions:**

The scientific production of Danish medic PhD-graduates was mainly produced around the time of PhD-graduation. After obtaining the PhD-degree the scientific production declines suggesting that scientific advance fails and resources are not harnessed.

## Background

The Doctor of Philosophy (PhD) is a common and universal education program in research. A priori, medical doctors achieve academic skills through the PhD in order to perform studies in basic and clinical trials of science. The outcomes of these are hoped to improve the care in humans suffering from curable or incurable diseases and to prevent their occurrence as well.

Although this well-meant strategy is underlying research training for medical doctors, the PhD has also become a necessary entry ticket for registrar training in popular clinical specialties. For instance, the popularity of clinical training in cardiology, endocrinology, gynecology and obstetrics, and plastic surgery is immense in Denmark, which creates wide competition among the applicants. In this context academic societies pick applicants based on research experience to a large degree. The salary costs in Denmark of PhD student can easily be 50.000 euros a year. Despite extensive academic productivity, harsh tongues may argue that the PhD is simply an instrument to get specialist training and question whether MDs achieving a PhD actually continue their academic activity after entering a clinical specialist training program. Previous studies suggest that more MDs do a PhD [1] and also that the majority publish peer- reviewed papers adhering to the PhD-graduation [2]. However, it remains unknown whether the PhD-program for medical doctors results in a continuing research production.

In order to investigate these issues, we examined Danish MDs achieving a PhD in Denmark to elucidate their postgraduate research activity. We used data from all Universities in Denmark from 1995 to 2013 and assessed individual academic productivity through systematic searching in the PubMed database.

## Methods

### Data sources

The University of Copenhagen, Aarhus, and Odense in Denmark gave permission to investigate their registries of medical doctors enrolled in their respective PhD-programmes and graduating between 1995 and 2013. These three Universities represent the entire country and are all state- run. No private universities educate MD-PhDs in Denmark. The three registries hold information on name, age, departments responsible for the research, dates of the PhD start (not for Aarhus, here we only had age at graduation), submission time of the PhD thesis, and graduation, respectively. In addition, titles of the PhD projects were available for each university over the period.

### Study design

Only PhD students with a precedent medical degree (MD) were included. This meant in practice that PhDs in internal medicine including general practice, surgery and basic science were investigated. Those who did not graduate before June 2013 were excluded. The MD is an integrated part of the medical education in Denmark. We excluded PhD students, who were guest visitors at the selected universities, and originally were enrolled at a foreign university. Students with a previous PhD in another field of science, and MPhil were excluded, too.

### Research productivity

PhD productivity was examined through data searches of online published papers in the PubMed database per individual PhD student, retrospectively. All published papers per individual were recorded and entered into a database. We included papers until August 2013 by conducting manual searches. If duplicate author names existed, published papers belonging to the author from the registry was determined according to the information of publication date, field of research, address of the author and research group and title of the PhD thesis. If duplicity of author names persisted, a minimum of two authors discussed the case and made a decision, or the authors were contacted if the distinction remained unclear. The search method was as broad as possible and therefore constructed only on family name followed by the first letter of the first name. Papers written by all relevant PhD graduates were registered. All papers included date of publication, rank of authors, journal name and title.

### Objectives

The primary outcome was post-PhD research inactivity, measured through the endpoint of a full year (365 days in a row irrespective of calendar years) without a peer-reviewed publication in PubMed. As a secondary objective we examined factors associated with inactivity.

### Statistics

Continuous variables were calculated as medians and inter-quartile ranges (IQRs). Proportions were presented as percentages (%). We calculated average numbers of publications per post- graduate time as well as by total time since first publication. We plotted the absolute number of publication as a function of time (time of PhD graduation set as index data). Time to event analyses were used to examine continuous research activity and this was illustrated using the Kaplan-Meier plot. The log-rank test was used to test for difference between the three universities. Logistic regression modelling was used to examine factors associated with inactivity incorporating pre-specified covariates as: sex, age, medical specialty, university, calendar year and distinction between surgery or medicine. A *P*-value of less than 0.05 was pre-specified as being significant.

Sensitivity analyses were undertaken in order taking into account of potential confounders and effect modifiers. Therefore, inactivity was extended to 2 years, publication was constrained to being first or last author and finally all publications in Danish medical journal was excluded, respectively. Missing data were not an issue since all citizens in Denmark have a citizen personal identification (CPR) number. In case the date of the PhD graduation was missing it was interpreted as a failure to accomplish the PhD title.

## Results

### Baseline characteristics

**Fig. 1:**
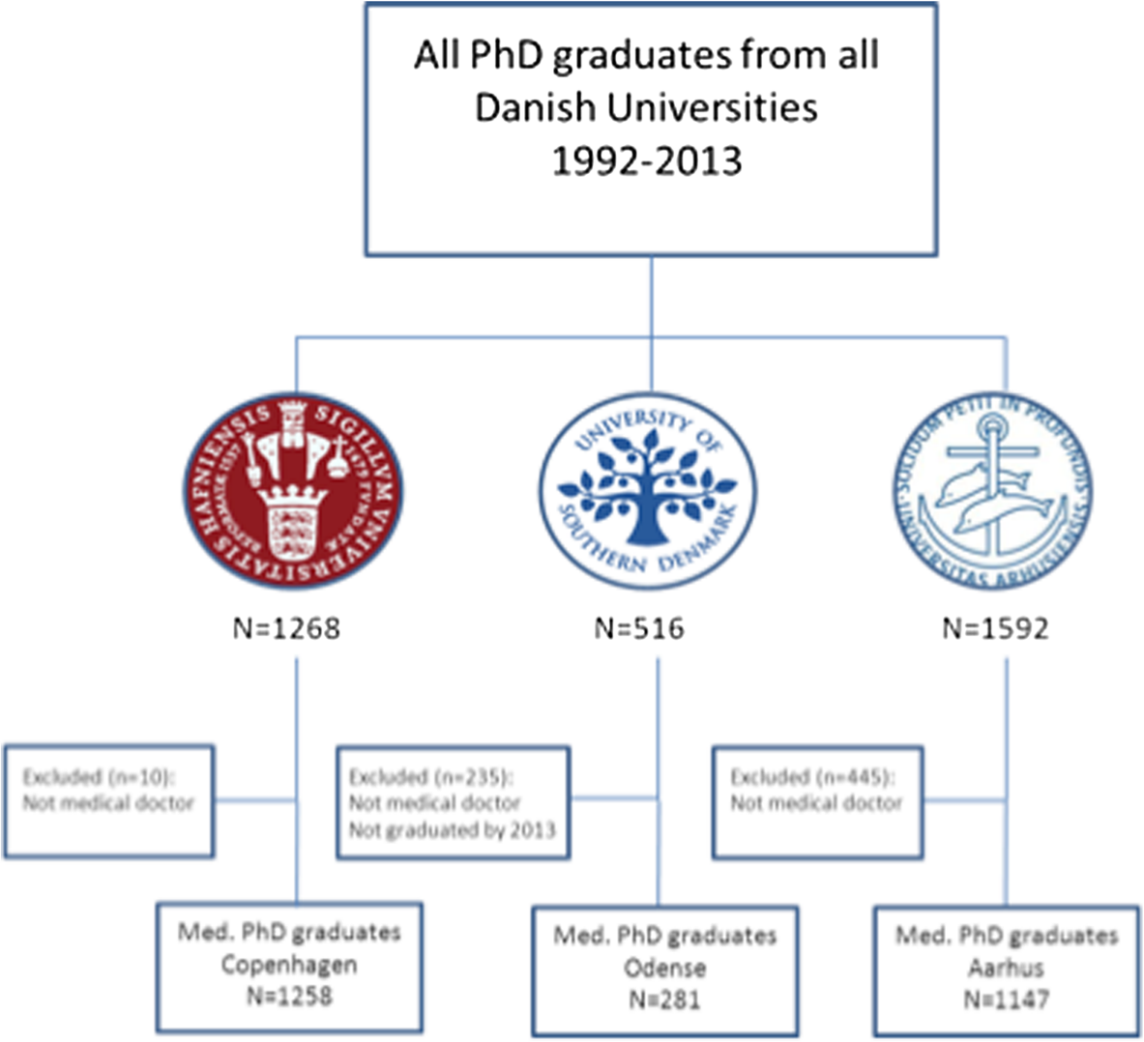
Study design

Figure 1 shows the selection process. A total of 2686 PhD graduates were included in our study; 1258, 281, and 1147 persons from Universities of Copenhagen, Southern Denmark, and Aarhus, respectively. Table 1 shows the characteristics of the PhD graduates. Overall, women were represented in higher numbers than men and the age was similar across the universities.

**Table 1:** Baseline characteristics of Danish PhD graduates

	Total	University of Copenhagen	University of Southern Denmark	University of Aarhus
N	2686	1258	281	1147
Female gender, N (%)	1424 (53.0 %)	694 (55.2 %)	134 (47.7 %)	599 (52.3 %)
Median age, years (IQR)	35 (32–38) ^b^	33 (31–36)	33 (31–36)	36 (34–49) ^a^
Year of graduation, N (%)				
1995–1999	401 (14.9 %)	203 (16.1 %)	40 (14.2 %)	158 (13.8 %)
2000–2004	717 (26.7 %)	300 (23.9 %)	92 (32.7 %)	325 (28.3 %)
2005–2009	799 (29.8 %)	389 (30.9 %)	74 (26.3 %)	336 (29.3 %)
2010–2013	769 (28.6 %)	366 (29.1 %)	75 (26.7 %)	328 (28.6 %)
PhD specialty, %				
Medicine	87.3 %	89.1 %	89.3 %	84.8 %
Surgery	12.7 %	10.9 %	11.7 %	15.2 %
Median duration of PhD, y (IQR)	4.0 (3.5–4.6)	4.0 (3.5–4.6)	4.3 (3.7–5.1)	---
Most productive departments, N (%)				
Cardiology	294 (11.0 %)	151 (12.0 %)	24 (8.9 %)	119 (10.4 %)
Endocrinology	273 (10.2 %)	117 (9.3 %)	36 (13.3 %)	120 (10.5 %)
Neurology	189 (7.1 %)	94 (7.5 %)	8 (3.0 %)	87 (7.6 %)
Gynecology/obstetrics	134 (5.0 %)	73 (5.8 %)	6 (2.2 %)	53 (4.6 %)

^a^age at graduation

^b^calculated including the Aarhus data, where the age is at graduation and not at enrollment

**Table 2:** Follow-up time and number of publications

	Total	University of Copenhagen	University of Southern Denmark	University of Aarhus
Median post-graduate follow-up, y (IQR)	6.9 (3.0-11.7)	6.6 (3.0-11.5)	8.0 (3.0-12.1)	6.9 (2.9-11.7)
Median no. of total publications (IQR)	9 (5–18)	11 (6–19)	11 (5–19)	8 (4–15)
Median no. of publications post-graduate (IQR)	7 (3–14)	5 (2–11)	11 (5–19)	8 (5–16)
Median no. of total publications per year (IQR)	1.12 (0.61-1.99)	1.17 (0.66-2.04)	1.02 (0.60-1.99)	1.05 (0.55-1.91)
Median no. of post-graduate publications per year (IQR)	1.32 (0.58-3.01)	1.09 (0.43-2.29)	1.92 (1.00-4.14)	1.60 (0.70-3.65)

### Research productivity

**Fig. 2:**
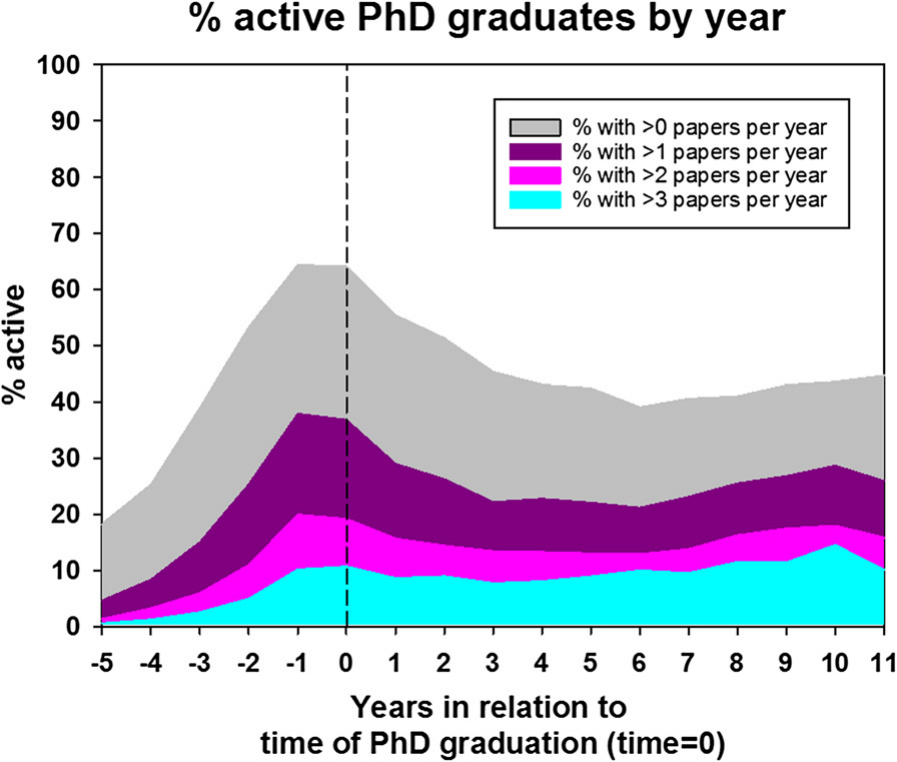
Percent of PhD graduates with >0, >1, >2, or >3 published papers per year as a function of time in relation to time of PhD-graduation (time 0)

**Fig. 3:**
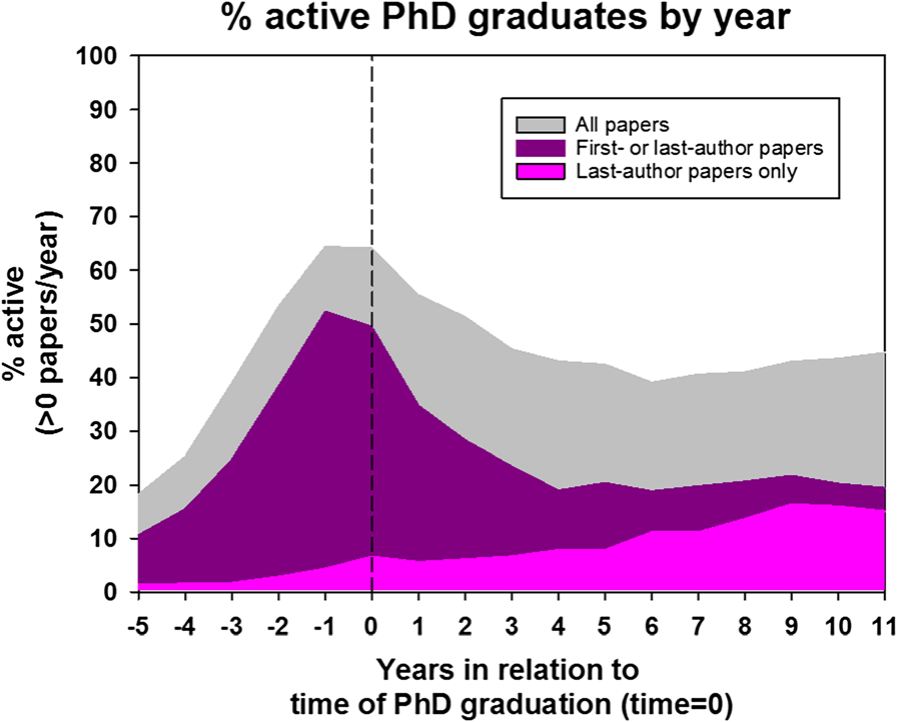
Percent of PhD graduates with an annual published paper, an annual first- or last-author paper, and a last-author paper as a function of time in relation to time of PhD-graduation (time 0)

The median follow-up time was 6.9 years (IQR, 3.0 to 11.7 years). Table 2 shows the median number of publications per PhD graduate. The median number of publications post-graduation was 7 (IQR, 3 to 14) corresponding to 1 paper per year. A total of 3.4 % of the PhD graduates had no publication after they graduated from the PhD program, 40 % had 5 or less, and 90 % had 30 or less. The relation between publications and time of PhD graduation is illustrated by Fig. 2, which shows that the majority of the publications are closely related to the period between PhD enrolment and graduation. After the index date, there was a decline in number of publications, which was reversed at 6 years by a slight increment. Figure 3 shows the relationship between author-placement on paper and time from graduation. First-author papers were most frequent around PhD graduation whereas co-author papers remained fairly stable throughout follow-up, but increased slightly after 4 to 5 years. Last-author papers were rare, but became more frequent as time passed.

### Inactivity

**Fig. 4:**
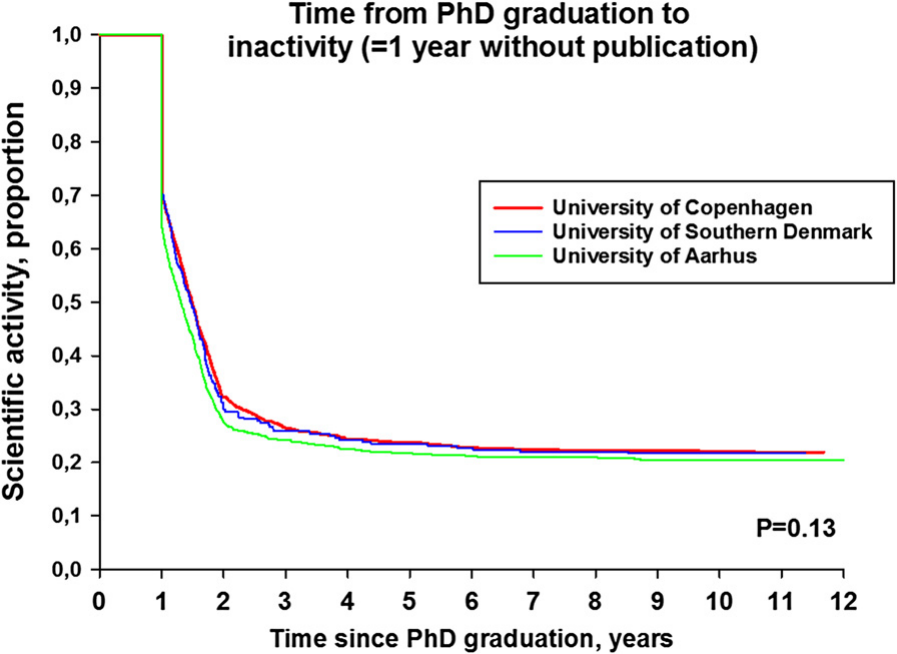
Time to inactivity (=1 year without publication) by Danish Universities. Differences between universities were tested with the Log-rank test and the *P* value was 0.13

Table 3 shows the characteristics of the PhD graduates who were inactive vs. active. Overall, 21.2 % of the graduates remained active over the whole follow-up, showing men more likely than women to remain active (23.1 % of men remained active vs. 19.6 % of women, respectively). Figure 4 shows time to inactivity demonstrating that 2/3 of PhD graduates became inactive after 2 years (depending on University of origin). Further decline over the follow-up period was observed without significant differences between the universities in unadjusted analyses (*P* = 0.13).

**Table 3:** Characteristics of Danish PhD graduates according to future research activity vs. inactivity

	Continued active	Inactive
N, % of total population	282 (10.5 %)	2404 (89.5 %)
Female gender, N (%)	118 (41.8 %)	1306 (54.4 %)
Median age, years (IQR)	34 (32–37)	35 (32–38)
PhD specialty, %		
Medicine	88.3 %	87.2 %
Surgery	11.7 %	12.8 %
University,%		
Copenhagen	36.5 %	48.0 %
Southern Denmark	9.2 %	10.6 %
Aarhus	54.3 %	41.4 %

A multivariable logistic regression model examined factors associated with inactivity. Gender was significantly associated with inactivity. Women were more likely to be inactive (adjusted odds ratio (OR); 1.59 (95 % confidence interval[CI] 1.24–2.05, *p* < 0.0001). Further, age and field of research (medicine or surgery) was not associated with inactivity (OR 1.02 per 1-year increment in age; 95 % CI 0.99–1.05 and OR for medicine referenced by surgery; 0.73, 95 % CI 0.50–1.07, *p* = 0.4). Similar to the unadjusted analysis we saw no difference in terms of activity/inactivity among the three Danish Universities (*p* = 0.2).

### Sensitivity analyses

We also examined inactivity at 2 years without a publication and the results did not change substantially (data not shown). Furthermore, for purposes of a sensitivity analyses we excluded papers, where the PhD graduate was not the first, second, or last author on the paper. This dropped the activity level even further (data not shown) and this was also the case if we excluded papers published in the Danish medical journal (data not shown).

## Discussion

This study examined the proportion of Danish medical PhD-graduates continuing research production measured through published papers. Data from all Danish universities were used and had a median follow-up of 7 years. Four main findings were found. First, a larger proportion of the PhD graduates were women, but characteristics of the individuals were similar across the three universities. Second, the majority of PhDs (two thirds) were inactive 2 years after graduation. Third, women were more likely to stop research than men. Fourth, last-author proportion increased after 4 to 5 years suggesting that being a research-supervisor/in chair of research projects took long time to develop.

Elsewhere, gender distribution among PhD graduates has been investigated. Two thirds of PhD-graduates at The University of Aarhus, Denmark were males in the period from 1993 to 1998 [2]. In contrast, contemporary analysis of inclusion within PhD programs from all PhD schools in Denmark suggested women to be in slight favour among the laureates. Furthermore, our results demonstrate a change in the gender distribution among medical PhD-graduates as well as a change in the gender distribution among physicians. The problem was also assessed by Kuehnle et al. who found that men constituted 2/3 of the PhD-graduates and students in the Swiss National MD-PhD program from 1992 to 2007 [3].

Approximately two thirds of the PhD-graduates became research inactive within two years after graduation in this study. Importantly, our results show that the majority of this measured activity is comprised by co-authorships and not first- or last-authorships. In addition, we relate last-author papers with a person who has evolved his research and is now in the role of supervisor/mentor. Our results show that such persons are few in numbers and that transition to last-author happens roughly 5–10 years post PhD-graduation. Furthermore the last-author proportion increased after 4 to 5 years suggesting that being a research-supervisor/in chair of research projects took long time to develop. Yet, still only 10–15 % of the MDs had last/first author publications at 8–12 years post-PhD graduation. We see this as a relatively low number and it probably represents those MDs who went on to take a fulltime or a part time research position (professors). It is indeed encouraging that we see this increase in senior authorships and this represents the long-term yield of the PhD education program in Denmark—yet, we also believe that this is a very low number. One could ask if it is rational to educate this many PhDs if only 10–15 % to some extend go on to build their own research groups/communities. Maybe a shorter program could be sufficient and create more researcher friendly programs for those with a special interest. Overall, our results contrast the findings of Jørgensen et al., who concluded that Danish PhD graduates in the years 1995–1997 remained productive after obtaining the degree [4]. In accordance with our results, Brass et al. investigated the career-path of several MD-PhDs over 40 years. They found that only one third of the questioned devoted more than 3/4 of their time to research [5].

The low degree of researchers having both a MD and PhD continuing to be research productive may also be explained by local factors. For instance, since February 2008 physicians from Denmark who have initiated their residency have a time limit of 5 years to achieve internship in either surgery or intern medicine. If the physician is unsuccessful, specialisation in Denmark is no longer possible according to the Danish health authority [6]. Stronger competition to achieve a specialist position over a constrained time horizon is implied by the increment in flow of PhD-graduates in Denmark. PhD-students from 2003 to 2013 has doubled demonstrated through annual increments [1]. Second, a substantial part of the PhDs will start clinical training following PhD-graduation reducing the probability of research continuation. In accordance; Whitcomb raised this as a central issue with the PhD-program and suggested restructuring of the PhD program [7]. Although reported elsewhere [8] that PhDs wish research as an important part of their future job description, our data could not confirm such a trend. However, a comparison between non-PhDs and PhDs was undertaken by Merani et al. and showed that volume and impact of research activity were greater for the PhD-graduates [9].

Factors associated with research inactivity suggested gender differences, as women were more likely than men to stop researching. This issue has been assessed in several studies. Lelièvre et al. concluded that male gender predicts publishing among pharmacists [10]. The gender gap has further been analysed by Kaufman et al. among physical therapy faculty members. They found similar results; male gender was a positive predictor for publication [11].

Our results suggest that future scientific yield from researchers with a medical background and the PhD is far from optimal. In order to improve productivity in the future, Whitcomb [7] suggested that the PhD program should be reformed through demands of higher scientific yields; also suggested by Olesen [12].

The external validity of our results is hard to say since differences between PhD schools worldwide are evident. However, the clinical work profile for most specialists are similar in western medicine, which could indicate that our results can be extrapolated to other countries if underlying reasons for not continuing research are lack of time or simply reduced competition as soon the PhD laureate achieve a consultant post. In Denmark, at least, the clinician does not have a strong incentive nor allocated time to continue doing research when he or she has secured a specialty training position (typically 5 years as fellow-in-training). Hence, research is conducted on their spare time or by taking leave from the fellowship (which very few trainees do). Nevertheless, the results here deserve to be replicated in other national based investigations, which may enhance the validity found here.

The current study had several limitations. The scientific yield of the PhD was evaluated retrospectively through manual searches in the PubMed database. Furthermore, this chosen search strategy was not validated. Another caveat was that searching in other databases such as Embase was not performed. Individual benefits from the PhD education, which were immeasurable, were not evaluated and may have intangible effects on these future medical doctors work as clinicians and their approach to medicine. However, a better understanding of clinical studies and statistical insight for the individual PhD graduate may conversely not contribute to better treatment for the overall population. Lack of detailed demographic data about the PhD-graduates was also a downside of our study. This means for example that we did not have access to personal information of PhD laureates being married after graduation. For instance, we had no information of the education level of the parents to the PhD graduates including a PhD or their social class. In addition we did not have access to personal covariates and career choice, which may have been explanatory for research activity. For instance, leave due to pregnancy and career choice of an academic or clinical pathway were all, unavailable. Accessing the latter may even have made a sharper statement of inactivity among clinicians with a PhD. We did not examine sub-groups of research according to type of research as these data were not readily available to us. An extended margin for research activity equating 3 or 4 years may also have increased the proportion of active researchers, but we still think that achieving funding and academic skills after 3 to 4 years of inactivity is difficult. During these years after PhD-graduates, the MD has many competing interests (especially clinical fellowship), but we believe that the system should be better at incentivising the graduates in continuing their research. Our results especially point to women as being more susceptible to this early decline in research productivity, which may be expected as child birth, clinical training, maternity leave and other competing interests are presenting themselves. We believe that funding mechanisms and research groups could be much better at holding the PhD graduates engaged during these straining years if the goal is to keep MDs research-active and hence help make patients and the future of medicine better. Eventually, we acknowledge that the MD PhD combination in Denmark may differ from countries elsewhere underscoring that the generalisability of the results remains elusive.

## Conclusion

In conclusion, we found that the majority of PhD graduates in Denmark stop their research activity shortly after being a laureate. Among those who remain active, being a co-author instead of a lead or a senior author mainly comprises this activity.
